# The Clinical and Biological Characterization of Triple A Syndrome (Allgrove Syndrome) in 12 Pediatric Patients

**DOI:** 10.7759/cureus.109586

**Published:** 2026-05-25

**Authors:** Ilham Bouarab, Fatimazahra Yakine, Fatima Zahra Alaoui-Inboui, Anas Abouelkheir, Zineb Hammoumi, Farida Jennane, Bouchra Slaoui

**Affiliations:** 1 Department of Paediatrics 2, Abderrahim Harouchi Mother and Children University Hospital, Casablanca, MAR; 2 Department of Paediatric Surgery, Abderrahim Harouchi Mother and Children University Hospital, Casablanca, MAR

**Keywords:** achalasia, adrenal insufficiency, alacrima, allgrove syndrome, pediatric, triple a syndrome

## Abstract

Introduction

Allgrove syndrome, also known as triple A (3A) syndrome, is a rare autosomal recessive disorder characterized by the association of achalasia, alacrima, adrenal insufficiency, and neurological manifestations. It is caused by the functional impairment of the nucleoporin ALADIN due to mutations in the AAAS gene. Data from African countries remain limited. We present the clinical profile, biochemical findings, management, and outcomes of 12 children with triple A syndrome who were admitted to a Moroccan tertiary care hospital.

Methods

This retrospective cohort study included 12 children diagnosed with Allgrove syndrome and followed in the Pediatric Endocrinology Department of Abderrahim Harouchi Mother and Child Hospital, Ibn Rochd University Hospital, Casablanca, Morocco, over a period of 14 years (January 2012 to January 2026). Diagnosis was based on at least two components of the clinical triad, supported by biological and radiological findings. Clinical, biological, radiological, and follow-up data were collected from the patients’ medical records.

Results

The median age at diagnosis was 4.3 years (interquartile range (IQR): 3.5-6). Adrenal insufficiency was the presenting feature in all patients. Alacrima was present in all cases and represented the earliest symptom, with a median age of onset of one year (IQR: 0-3). Dysphagia was observed in seven (58.3%) patients, with a median age at onset of four years (IQR: 3-5). All patients received hydrocortisone replacement therapy. Mineralocorticoid deficiency was identified in two (16%) cases. Four patients underwent Heller myotomy with fundoplication, with favorable outcomes.

Conclusions

Alacrima was the earliest and most consistent clinical feature in our cohort, while adrenal insufficiency represented the main presenting manifestation. Prompt recognition of these findings is crucial to facilitate early diagnosis and improve patient outcomes, particularly in resource-limited settings.

## Introduction

Allgrove syndrome, also known as triple A (3A) syndrome, is a rare autosomal recessive neuroendocrine disorder, with an estimated prevalence of approximately one case per 1,000,000 individuals [1]. It is characterized by the association of alacrima, achalasia, and adrenal insufficiency. In addition to this classic triad, approximately two-thirds of patients develop neurological manifestations involving the central, peripheral, and autonomic nervous systems, leading some authors to refer to the condition as 4A syndrome. Additional clinical manifestations have also been reported, including xerostomia, dental caries, palmoplantar hyperkeratosis, dysmorphic facial features, gait disturbances, and delayed puberty, reflecting the multisystemic nature of the disorder [1,2,3].

Genetically, Allgrove syndrome is associated with mutations in the AAAS gene, located on chromosome 12q13, which encodes the nuclear pore complex protein ALADIN, identified in approximately 90% of cases. This protein plays a key role in nucleocytoplasmic transport, particularly of proteins involved in DNA repair. Its dysfunction impairs transport mechanisms, increasing cellular susceptibility to oxidative stress and leading to selective tissue degeneration [4]. Since its first description in 1978, several hundred cases of Allgrove syndrome have been reported in the literature, with marked phenotypic variability. To the best of our knowledge, this study represents one of the largest case series of Allgrove syndrome reported in Morocco [5]. This study aims to describe the clinical, biochemical, evolutionary, and therapeutic characteristics of 12 children with Allgrove syndrome, followed at the pediatric endocrinology unit of the Abderrahim Harrouchi Children’s Hospital.

## Materials and methods

We conducted a retrospective cohort study involving children diagnosed with Allgrove syndrome and followed at the pediatric endocrinology unit of the Abderrahim Harrouchi Children’s Hospital. A total of 12 patients diagnosed over 14 years, from January 2012 to January 2026, were included.

Inclusion criteria comprised all patients under 15 years of age diagnosed with Allgrove syndrome. Patients older than 15 years and those with incomplete medical records were excluded. Data were collected retrospectively from inpatient hospitalization files, outpatient follow-up records, laboratory reports, imaging studies, and endoscopic examinations, and entered into a structured database using Microsoft Excel. Statistical analysis was performed using the same software, and results were expressed as medians with interquartile ranges (IQRs) for continuous variables and as counts and percentages for categorical variables.

The clinical diagnosis was based on the presence of at least two components of the classical triad. Alacrima was assessed clinically and confirmed using a positive Schirmer test, defined as tear production of less than 10 mm over five minutes. Symptoms suggestive of achalasia were evaluated through clinical history and confirmed by upper gastrointestinal endoscopy and barium esophagography. Adrenal insufficiency was confirmed biochemically by a morning (8 a.m.) serum cortisol level below 5 µg/dL, associated with elevated plasma adrenocorticotropic hormone (ACTH) levels exceeding twice the upper limit of normal. Mineralocorticoid deficiency was defined by elevated plasma renin levels above age-specific reference ranges.

Neurological and dysautonomic involvement was assessed through a detailed clinical history and a comprehensive neurological examination, including evaluation of motor, sensory, and autonomic functions. In the absence of suggestive clinical signs, no additional investigations were performed. Genetic testing was not performed due to limited access to molecular diagnostic facilities and financial constraints.

Epidemiological (sex, age at diagnosis, consanguinity, family history), clinical, and biological data were collected. Phenotypic characteristics, including the age of onset of major features (alacrima, achalasia, adrenal insufficiency, and neurological involvement), were also analyzed. Therapeutic management and clinical outcomes during follow-up were evaluated. The study was conducted in accordance with ethical standards, and patient data were anonymized.

## Results

The retrospective analysis of medical records from 12 children belonging to 11 different families showed that the median age at diagnosis was 4.3 years (IQR: 3.6-6 years). A male predominance was observed, with a sex ratio of 1.4 (seven males, five females). Parental consanguinity was found in five (41.7%) cases. Two patients (16.7%) had a history of sibling death in a context suggestive of undiagnosed Allgrove syndrome: one case presented with cutaneous hyperpigmentation associated with alacrima, while the other died in the setting of meningitis associated with alacrima. The median follow-up duration was 3.9 years (IQR: 1.25-6.7 years).

The most common presenting complaint was asthenia with cutaneous hyperpigmentation, observed in 10/12 (83.3%) patients. In two (16.7%) cases, the diagnosis was revealed by seizures secondary to hypoglycemia related to adrenal insufficiency. Alacrima was the earliest symptom reported by parents during early childhood, with a median age of onset of one year (IQR: 0-3 years). Half of the patients (n = 6, 50%) presented with alacrima from birth. Ophthalmological examination confirmed decreased tear production in all patients, with a Schirmer test ≤ 5 mm. Artificial tear replacement therapy was initiated in all cases. Dysphagia was present in 7/12 (58.3%) patients at the time of diagnosis, associated with recurrent vomiting. The median age of onset was four years (IQR: 3-5 years). One patient developed dysphagia at the age of seven years, while four (33.3%) patients had no dysphagia during the study period.

**Figure 1:**
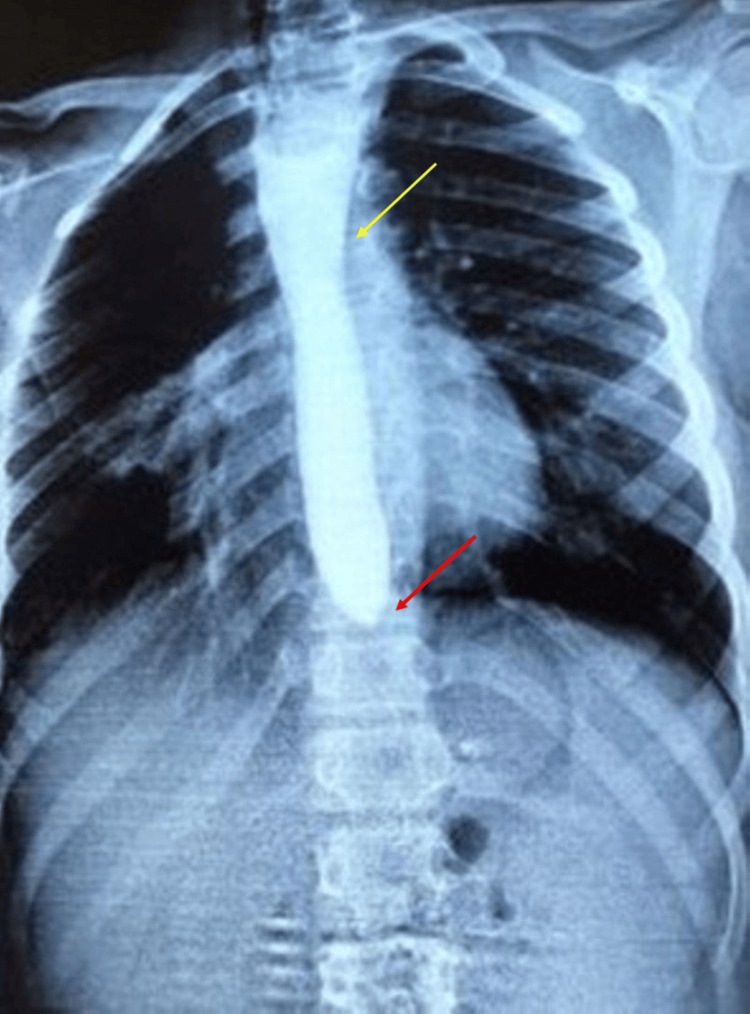
Barium esophagography (frontal view) in a patient from this series The image shows marked esophageal dilatation with prolonged contrast stasis (yellow arrow), absence of effective peristalsis, and distal “bird’s beak” narrowing (red arrow), consistent with megaesophagus suggestive of achalasia

**Figure 2:**
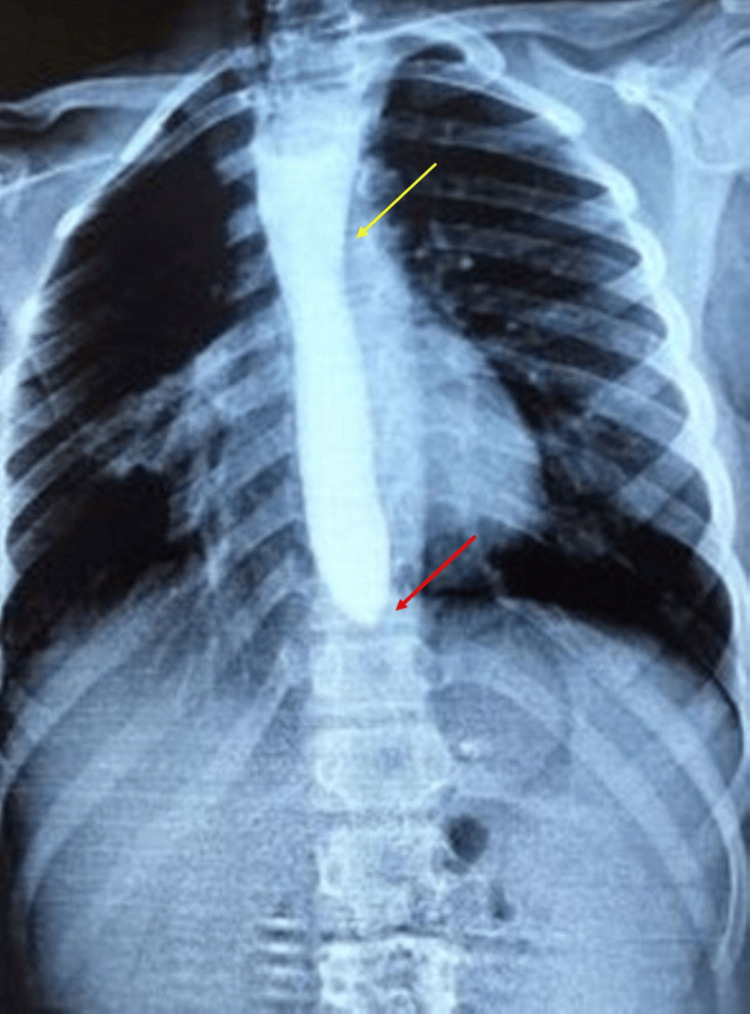
Barium esophagography (lateral view) in a patient from this series The findings confirm esophageal dilatation with contrast retention (yellow arrow) and distal “bird’s beak” tapering (red arrow), consistent with achalasia

Barium esophagography and upper gastrointestinal endoscopy were performed in all patients, revealing megaesophagus in the seven symptomatic patients as well as in one asymptomatic patient (Figures 1, 2). Esophageal manometry was performed in only two patients, demonstrating type III achalasia in one case and type II achalasia in the other. In the remaining patients, manometry could not be performed due to the unavailability of the procedure in our hospital.

Regarding management, four patients underwent Heller myotomy combined with Nissen fundoplication, with favorable clinical outcomes. One patient is currently awaiting surgery, while three patients with mild dysphagia are being managed conservatively. All patients presented with clinically evident adrenal insufficiency, confirmed biochemically by low serum cortisol levels, with a median of 4 µg/dL (IQR: 3.3-4.5 µg/dL), associated with elevated plasma ACTH levels, with a median of 731.5 pg/mL (IQR: 236-2001 pg/mL). None of the patients required a Synacthen stimulation test for diagnostic confirmation.

Most patients (n = 10, 83.3%) had a normal mineralocorticoid axis, while two (16.7%) exhibited mineralocorticoid deficiency, evidenced by hyponatremia, hyperkalemia, low aldosterone levels, and elevated plasma renin activity. Hydrocortisone replacement therapy was initiated in all patients at a median dose of 15 mg/m²/day (IQR: 14-16 mg/m²/day), administered in three divided doses. Therapeutic education was provided to both parents and patients, focusing on the recognition of adrenal crisis symptoms (vomiting, malaise, hypotension), the need to increase hydrocortisone doses during stress (fever, infection, surgery), and the use of intramuscular hydrocortisone injection in emergencies when oral administration is not possible (emergency kit). Fludrocortisone was prescribed in two patients at a dose of 0.1 mg/day. Artificial tear replacement therapy was initiated in all cases.

All patients were followed up regularly every three to six months, with assessment of growth parameters and growth velocity. Clinical monitoring for signs of overtreatment (excessive weight gain, decreased growth velocity, hypertension) and undertreatment (asthenia, anorexia, weight loss, hyperpigmentation, nausea/vomiting, hypotension) was systematically performed. Biological follow-up included serum sodium and potassium levels to assess electrolyte balance, as well as plasma renin activity and ACTH levels to guide treatment adjustments.

Regarding other clinical manifestations, one patient presented with severe dental caries associated with palmoplantar hyperkeratosis. None of the patients in the cohort exhibited neurological, dysautonomic, or cardiovascular manifestations. All patients in this series received coordinated multidisciplinary care involving a pediatric endocrinologist, ophthalmologist, pediatric surgeon, and neurologist. Table 1 summarizes the clinical and biological characteristics of the 12 cases included in this study.

**Table 1:** Clinical and biological characteristics of patients with Allgrove syndrome included in this series (n = 12) ACTH: adrenocorticotropic hormon; M: male; F: female; N: no; Y: yes; AI: adrenal insufficiency; –: not identified

Cases	Sex	Presenting feature/age (years)	Age at last consultation (years)	Alacrima/age at onset (years)	Achalasia/age at onset (years)	AI/age at onset (years)	Neurological dysfunction/age at onset (years)	Family history/parental consanguinity	Basal cortisol (µg/dl)	ACTH (pg/ml)	Plasma renin (mUI/l)	Follow-up duration (years)
1	M	AI/6	13.3	Y/0	Y/4	Y/6	N	N/N	4.5	120	230	7.3
2	M	AI/4	10.1	Y/3	Y/4	Y/4	N	Y/Y	5	207	19.2	6.1
3	F	AI/4.2	9	Y/2	Y/3	Y/4.2	N	Y/Y	3.5	220	0.6	4.8
4	M	AI/3	6	Y/3	Y/3	Y/3	N	N/N	6	2000	0,5	3
5	F	AI/6	15.2	Y/0	N/-	Y/6	N	Y/Y	4.2	252	21.7	9.2
6	M	AI/1.6	11.6	Y/0	N/-	Y/1.6	N	N/Y	10	463	11.2	10
7	M	AI/6	12	Y/4	Y/5	Y/6	N	N/Y	4	1000	1.3	6
8	F	AI/3	4.8	Y/0	Y/2	Y/3	N	Y/N	3.4	4130	267	1.8
9	M	AI/4.3	5	Y/2	N/-	Y/4.3	N	N/N	3.3	1680	0.9	0.7
10	M	AI/5.3	5.9	Y/0	N/-	Y/5.3	N	N/N	2	400	1.12	0.6
11	F	AI/7.2	9	Y/3	Y/6	Y/7.2	N	N/N	4.14	2002	15.2	1.8
12	F	AI/4.1	4.2	Y/0	N/-	Y/4.1	N	N/N	0.15	2300	6.7	0.1
Laboratory reference range									31.18-97.15	7.40-64.30	2.8-39.9	

## Discussion

Allgrove syndrome is a rare autosomal recessive genetic disorder characterized by marked phenotypic variability, leading to delayed diagnosis, particularly in incomplete forms [1]. In populations with high consanguinity rates, particularly in the Middle East and North Africa, a higher frequency of the disease is observed. The identification of founder mutations in the AAAS gene suggests a founder effect in these regions [6]. In our series, parental consanguinity was observed in 41.7% of cases. Although this rate is higher than that reported in some European cohorts (approximately 20-30%) [7], it remains lower than that observed in populations with high consanguinity rates. Indeed, consanguinity rates approaching 95% have been reported in certain African cohorts, often associated with near-constant homozygosity and founder mutations in the AAAS gene [3,8].

In our series, the median age at diagnosis was 4.3 years, reflecting a moderate diagnostic delay. This finding is consistent with findings from the literature, which demonstrate considerable variability in the age at diagnosis of Allgrove syndrome [3,6]. This variability can be partly explained by the marked phenotypic heterogeneity of the syndrome, both in terms of age of onset and severity of clinical manifestations. Furthermore, the progressive course of the disease suggests the involvement of a degenerative process in its pathophysiology. Incomplete or atypical forms may progressively evolve toward the classical phenotype over time, which may contribute to delayed diagnosis [3].

Additional factors may also influence the timing of diagnosis, including the delay between the onset of initial symptoms, particularly alacrima, and its recognition and medical evaluation, as well as the level of awareness among clinicians regarding this rare condition. Finally, genetic and sociocultural factors, particularly high consanguinity rates, limited access to healthcare, and delayed medical consultation, may also contribute to delayed diagnosis.

In our series, alacrima was present in 100% of patients, confirming its role as a cardinal feature of Allgrove syndrome, reported in more than 90% of cases in the literature [9,10]. It is often observed by parents from early childhood, yet it nevertheless remains frequently overlooked, as it does not always prompt medical consultation [1]. Its pathophysiology remains incompletely understood; however, dysfunction of the autonomic nervous system at the level of the lacrimal glands has been suggested [11]. The Schirmer test remains the gold standard for confirming the diagnosis, while management is mainly symptomatic, including artificial tears and lubricating eye drops. Furthermore, alacrima is a rare manifestation described in a limited number of congenital disorders [12]. Its identification in children should prompt a diagnostic workup for adrenal insufficiency and dysphagia.

Regarding achalasia in Allgrove syndrome, its pathophysiology is thought to involve damage to the myenteric plexus, characterized by degeneration of inhibitory neurons responsible for relaxation of the lower esophageal sphincter. This alteration leads to an imbalance between excitatory and inhibitory pathways, resulting in loss of esophageal peristalsis and incomplete sphincter relaxation. Mutations in the AAAS gene may promote neuronal oxidative stress and dysfunction of the autonomic nervous system, suggesting a diffuse autonomic neuropathy as a common underlying mechanism [13,14].

The management of achalasia relies on several approaches. Pharmacological treatments, although of limited efficacy, mainly include calcium channel blockers such as nifedipine and nitrates, which help reduce lower esophageal sphincter pressure. Other therapeutic options include pneumatic dilation, generally used in selected patients with less severe symptoms or as a temporary measure; laparoscopic Heller myotomy combined with fundoplication, which remains the standard surgical treatment for significant or progressive achalasia; and peroral endoscopic myotomy (POEM), a less invasive alternative increasingly used in selected cases [1].

In this cohort, achalasia was present in 66.6% of patients, a rate consistent with previously reported series, as achalasia represents one of the major manifestations of Allgrove syndrome, described in approximately 60-90% of cases [7,15]. These findings confirm that achalasia constitutes, after alacrima, one of the major manifestations of Allgrove syndrome, although its frequency may vary depending on genetic factors and population characteristics. Adrenal insufficiency is one of the most frequent and severe manifestations of Allgrove syndrome and represents the leading cause of mortality in undiagnosed patients. It most commonly results from ACTH insensitivity [3].

However, the mechanisms responsible for the selective tissue involvement observed in Allgrove syndrome remain incompletely understood. Although ALADIN dysfunction is known to contribute to adrenal insufficiency, its precise role in adrenal cell function and ACTH signaling pathways, particularly involving MC2R and SCARB1, has not yet been fully elucidated [16]. In this cohort, clinical manifestations of adrenal insufficiency represented the initial presentation and led to the diagnosis of Allgrove syndrome in all patients. All patients exhibited cutaneous hyperpigmentation, either generalized or localized. In addition, two families reported a history of sibling death in a context suggestive of undiagnosed adrenal insufficiency.

The age at diagnosis of primary adrenal insufficiency was variable, with a median of 4.3 years. However, all patients developed symptoms within the first decade of life, and more than half (58.3%) before the age of five. These findings are consistent with those reported in the literature [1,3]. Mineralocorticoid deficiency, related to involvement of the zona glomerulosa, remains a rare manifestation of Allgrove syndrome. Its prevalence varies across studies and is estimated at around 15% [3,7], which is consistent with our findings, where only 16% of patients exhibited such a deficiency. Our cohort did not exhibit significant neurological or dysautonomic manifestations. This may be explained by the delayed onset of neurological involvement during the course of the disease, as well as by the fact that our cohort consisted exclusively of pediatric patients.

This study has several limitations, foremost being the small sample size, which is expected given the rarity of Allgrove syndrome. In addition, genetic confirmation through AAAS gene sequencing was not performed due to its unavailability in our setting, which constitutes another limitation. However, the diagnosis was based on strict clinical criteria, notably the characteristic triad, combined with biological findings consistent with ACTH-resistant primary adrenal insufficiency.

## Conclusions

This series highlights the early onset of alacrima as a key presenting feature, contrasting with the delayed recognition of the other components of the triad in Allgrove syndrome. Early multidisciplinary management, including regular ophthalmologic follow-up, appropriate hormone replacement therapy, and surgical treatment of achalasia when indicated, was associated with favorable clinical outcomes in our cohort. These findings highlight the importance of heightened clinical awareness of triple A syndrome to facilitate earlier diagnosis and improve prognosis, particularly in resource-limited settings. They also support, where available, the use of genetic testing to confirm the diagnosis, with particular relevance for recurrence risk evaluation and genetic counseling.
